# US specificity of occasion setting: Hierarchical or configural learning?

**DOI:** 10.1016/j.beproc.2012.03.005

**Published:** 2012-07

**Authors:** Charlotte Bonardi, Craig Bartle, Dómhnall Jennings

**Affiliations:** School of Psychology, University of Nottingham, University Park, Nottingham NG7 2RD, UK

**Keywords:** Rat, Occasion setting, Hierarchical, Configural learning, Summation

## Abstract

Four experiments in rats examined whether occasion setters and target CSs play qualitatively different roles in occasion-setting discriminations. Two visual occasion setters, *A* and *B*, signalled reinforcement of two auditory target CSs, *x* and *y*, with sucrose and oil (*A*…*x* → *suc*, *B*…*y* → *oil*, *A*−, *B*−, *x*−, *y*−); in addition two transfer CSs *w* and *z* were paired with sucrose and oil (*w* → *suc*, *z* → *oil*). When *w* and *z* were substituted for *x* and *y* (*A*…*w*, *B*…*w*, *A*…*z*, *B*…*z*) more responding was observed when both stimuli had been paired with the same outcome (Experiments 1 and 3a). No effect was observed when two visual “pseudo-occasion setters”, *C* and *D* (paired with sucrose and oil in a trace relation to the US:*C*… → *suc*, *D*… → *oil*), were substituted for the occasion setters *A* and *B* (C…*x*, *D*…*x*, *C*…*y*, *D*…*y*; Experiments 2, 3b and 4). These results could not be explained in terms of Pavlovian summation: responding to combinations of Pavlovian CSs paired with same or different outcomes was either the same, or lower when both stimuli had been paired with the same outcome (Experiment 4). Implications of these results for theories of occasion setting and configural learning are discussed.

## Highlights

► More responding when occasion setter signals transfer CS signalling the same outcome. ► Similar responding when pseudo-occasion setter signals target CS signalling the same outcome. ► Less responding when transfer CS signals target CS signalling the same outcome. ► Results inconsistent with configural theory, consistent with hierarchical theory.

## 1 Introduction

Occasion setters are stimuli that signal that a conditioned stimulus (CS), that is otherwise without consequence, will be followed by an unconditioned stimulus (US); as a result the CS elicits a greater conditioned response (CR) when it is preceded by the occasion setter than when it is presented alone. Critically, this behaviour is independent of the Pavlovian properties of the occasion setter. For example, it is maintained after extinction of the occasion setter (e.g., Holland, 1989); moreover, animals can learn biconditional tasks in which *A* signals reinforcement of *x* and not *y*, while *B* signals the reverse (i.e. *A*…*x*+, *A*…*y*−, *B*…*y*+, *B*…*x*−; e.g., Asratyan, 1961). As *A* and *B* are equally associated with reinforcement and nonreinforcement, as are *x* and *y*, Pavlovian conditioning could not predict more responding to *x* on reinforced *A*…*x*+ trials than on nonreinforced *B*…*x*− trials, and so could not explain accurate performance on this task.

Evidence suggests that occasion setters act hierarchically on the CS–US association (e.g., Bonardi, 1996; Bonardi and Ward-Robinson, 2001; Rescorla, 1991a, 1991b; Swartzentruber, 1995) – for example, by facilitating the flow of activation between CS and US (e.g., Holland, 1983; see also Bouton, 1990; Bouton and Nelson, 1998; cf. Skinner, 1938). This *hierarchical* theory has some face validity: it seems natural to say that a CS predicts a US only on the specific occasions when it is signalled by the occasion setter (for example, a friend only becomes irritable at exam time). Nonetheless, little is yet known about precisely how occasion setters form and act (although see Rescorla, 1986, 1988). For example, some have suggested that there is an association between the occasion setter and the entire target CS → US association (Holman and Mackintosh, 1981; see also Bonardi, 1991, 1998; Swartzentruber, 1991). In support of this suggestion, Bonardi and Jennings (2009), trained animals on a biconditional discrimination in which *A* signalled that *x* would be followed by food whereas *y* would not (*A*…*x*+, *A*…*y*) while *B* signalled the opposite (*B*…*y*+, *B*…*x*−). Then *A* was paired with shock, and the amount of fear evoked by *x* and *y* assessed as a function of whether *x* and *y* were followed by food or not. Animals showed more fear after experiencing a pairing of *x* and food than a pairing of *y* and food – but showed more fear after a *nonreinforced* presentation of *y* than after a nonreinforced presentation of *x*. The authors argued that when *A* was paired with shock, the *x* → *food* and *y* → *no food* associations became paired with shock, producing the selective responding at test (see also Honey and Watt, 1998).

The hierarchical account assumes new principles in order to explain occasion setting, as it envisages that the occasion setter *facilitates* the association, rather than activating representations in the standard associative manner. But some argue that this approach is overly complex and that it would be better to adapt existing associative principles to account for occasion-setting effects. Such an approach has been adopted by a body of models broadly referred to as configural theories; these accounts are characterised by the assumption that compound cues are something more than the sum of their constituent elements (e.g. Brandon et al., 2000; Pearce, 1987, 1994; Rescorla, 1972, 1973; see also Honey and Ward-Robinson, 2001). For example, concurrent presentation of the occasion setter and target CS could be said to activate a configural stimulus representation that is distinct from the representations of its two constituent elements (e.g. Pearce, 1987). This configural cue will acquire associative strength during occasion-setting training, but will not be present when the occasion setter is presented alone. Thus extinction of the occasion setter would leave the associative strength of the unique cues intact, and the discrimination would be maintained – the key characteristic of occasion setting. Similarly, the biconditional discrimination referred to above, *Ax*+, *Ay*−, *Bx*−, *By*+ would be more accurately represented as a ***p***+, ***q*****−**, ***r*****−**, ***s***+ discrimination, where ***p***, ***q***, ***r*** and ***s*** are the configural cues corresponding to the ***Ax***, ***Ay***, ***Bx*** and ***By*** compounds, respectively; this would explain solution of the task (cf. Brandon et al., 2000).

There is now considerable evidence that configural accounts of this type can explain learning in a variety of circumstances, including occasion-setting discriminations (e.g. Williams et al., 1994). Nonetheless, in some situations a hierarchical description seems a more intuitively accurate description of the contingencies in operation: the friend who is irritable during exam time is still the same friend, and yet a configural description implies that the stimulus compound differs in an important way from its constituents, and so cannot accommodate such an intuition. One could perhaps apply the same intuition to behavioural paradigms in which the occasion-setting cue signals rather than accompanies the CS, or when it is an experimental context in which the CS is embedded. The aim of the present experiments is therefore to examine whether configural accounts are sufficient to explain all occasion-setting type discriminations.

One way of discriminating experimentally between hierarchical and configural explanations of occasion setting relates to *transfer*. This refers to the degree to which an occasion setter which has signalled reinforcement of its target CS with a particular outcome can control responding to a different, *transfer* CS, paired with either the same or a different outcome. The version of hierarchical theory outlined above may suppose that the transfer CS shares some common elements, CS_C_, with the target CS from the occasion-set association, and that likewise the transfer US shares common elements, US_C_, with the US of the occasion-set association. Thus the component of the transfer CS → US association that is represented by the CS_C_ → US_C_ link will be under control of the occasion setter – and the more common elements that are shared by the components of the two associations, the greater such control will be. In short, this account predicts that transfer will be more effective if the outcomes of the occasion setter and the transfer target are the same.

Configural theories, on the other hand, typically cannot anticipate this result, because performance is maintained by the associative strength of a unique cue produced by the combination of the occasion setter and CS – the outcome is not represented (but see e.g. Honey and Watt, 1999). Thus if the original CS is substituted by a transfer CS, responding will be maintained to the extent that the test compound is similar to the training compound – so that an occasion setter should be equally effective with a transfer CS, regardless of whether its outcome matches that of the occasion-setting discrimination or not.

## 2 Experiment 1

Rats were trained on a task in which an occasion setter *A* signalled that target CS *x* was reinforced with sucrose, and occasion setter *B* signalled that *y* was reinforced with oil. Animals were also trained with two transfer CSs, *w* and *z*, which were followed by sucrose and oil, respectively (see Table 1). According to hierarchical theory the occasion setters should promote responding more effectively to transfer CSs that signal the same outcome as the occasion setter; thus *A* should produce responding to *w* more than to *z*, whereas *B* should show the opposite pattern. Standard configural theory, in contrast, would not predict a difference between these two conditions because the outcome is not represented in the configural cue.

### 2.1 Materials and method

#### 2.1.1 Subjects

The subjects were 16 male hooded Lister rats with a mean *ad lib.* weight of 301 g (range = 280–330 g). They were deprived to 85% of their *ad lib.* weight before the start of the experiment, and were maintained at this level (with regular increments to allow for natural growth rate) by being fed a restricted amount of food at the end of each session; they were housed in pairs in plastic tub cages with sawdust bedding. The colony room was lit from 8 am to 8 pm; the subjects were tested during the light portion of the cycle.

#### 2.1.2 Apparatus

A set of four standard Skinner Boxes (supplied by Campden Instruments Ltd.) were used, each housed in a sound- and light-attenuating shell. Each box had three walls of sheet aluminium, a transparent plastic door as the fourth wall, a grid floor and an aluminium ceiling. One of the walls adjacent to the door contained a recessed food tray covered by a transparent plastic flap, 6 cm high × 5 cm wide that was hinged to the top of the food tray opening. Pushing this flap inward from its vertical resting position allowed subjects access to the food tray, and actuated a microswitch, and each switch closure was recorded as a single response; the flap automatically returned to its resting position when the rat removed its head from the food tray. The boxes were normally illuminated by a 2.8-W houselight, operated at 12 V, situated on the front wall directly above the food tray. 45 mg sucrose pellets (Noyes, New Hampshire) could be delivered to the food tray, as could deliveries of groundnut oil, which were delivered from a reservoir outside the chamber with a peristaltic pump. The reinforcers were either the delivery of 2 sucrose pellets, or of .3 ml of groundnut oil (Sainsbury's, UK), delivered by operating the pump for 160 cs. There were two visual stimuli; one was the pulsed illumination of two, 2.8-W jewel lights, both situated on the front wall, one to the right of the food tray and one to the left; these lights flashed 500 ms on alternated with 500 ms off; the second was provided by illumination of a 2.8-W bulb mounted inside the food tray (the traylight). There were four auditory stimuli: a 75-dB white noise, a 10-Hz 75-dB clicker, a 300-Hz 74-dB buzz and a 2-kHZ 75-dB tone; all were produced by Campden instruments noise and tone generators and delivered through a speaker mounted on the chamber wall. The floor comprised stainless-steel rods .5 cm in diameter and 1.5 cm apart. The boxes were controlled by a BBC microcomputer programmed in a version of BASIC (ONLIBASIC, written by Steve Channell).

#### 2.1.3 Procedure

##### 2.1.3.1 Pretraining

Subjects first received one 40-min session of magazine training in which sucrose pellets were delivered according to a VT-60 s schedule, and a second in which 5 presentations of oil were presented on a VT 300-s schedule. Animals that did not eat all the reinforcers in either session type received extra sessions until they had done so.

##### 2.1.3.2 Stage 1: positive patterning training

In sessions 1–16 all animals were trained on a positive patterning discrimination in which the two occasion setters *A* and *B* (jewel and traylight) signalled that the target CSs *x* and *y* (noise and click) would be reinforced; for half the animals the jewel lights signalled reinforcement of the click, and the traylight reinforcement of the noise; for the remaining subjects this arrangement was reversed. For half of each subgroup the click signalled sucrose and the noise oil, and for the remainder the reverse. Animals received six reinforced compound trials (6*A*…*x*+, 6*B*…*y+*), and 15 nonreinforced presentations of each stimulus alone (15*A*−, 15*B*−, 15*x*−, 15*y*−), giving a total of 72 trials per session. In this and all subsequent experiments the intertrial interval (ITI) was of a mean duration of 75 s (range 60–90 s), and all stimuli were of 10 s duration; on compound trials a 5-s trace interval separated occasion setter offset and target CS onset. The different types of trial were presented in a quasi-random order.

##### 2.1.3.3 Stage 2: transfer CS training

Animals were then given training with the two transfer CSs, *w* (the buzz) and *z* (the tone), while receiving further positive patterning training. For all animals *w* was paired with sucrose and *z* with oil. Sessions 17–20 were identical to those of the previous stage except that three of each of the two types of compound trial were replaced by three of each type of transfer CS trial (3*w*+, 3*z*+). Sessions 21–32 were identical to those of the previous stage except that *w* and *z* trials were given in addition to the remaining trial types, giving a total of 78 trials per session. Sessions 33–34 were identical to sessions 21–32 except that *w* and *z* were nonreinforced, in order to reduce responding to these stimuli, both so that transfer CS responding would be low enough for elevation to be detectable, and also because occasion setters do not transfer to CSs that have no history of nonreinforcement (e.g. Rescorla, 1985).

##### 2.1.3.4 Test

The first and second test sessions combined positive patterning training with test trials; however during testing it became apparent that there were too few test trials to produce a sufficiently large sample of behaviour, and so the third test omitted the positive patterning training trials. This testing technique was retained throughout all subsequent experiments.

###### 2.1.3.4.1 Tests 1 and 2

The first test sessions, 35–38 and 41–44, were identical to those of the positive patterning stage except for the addition of 9 additional test trials – three with one of the transfer CSs alone, three with it signalled by *A* and three with it signalled by *B* (e.g., 3*w*−,3*A*…*w*−, 3*B*…*w*−); the transfer CS was either *w* or *z* in the sequence *w*/*z*/*z*/*w*/*z*/*w*/*w*/*z*. Sessions 39–40 and 45–48 were retraining sessions (as sessions 21–32). The sessions of the second test, 49–56, were identical to positive patterning sessions except that the number of trials was adjusted so that an increased number of test trials could be included. Thus there were 12 of each nonreinforced trial (12*A*−, 12*B*−, 12*x*, 12*y*−), and 5 of each reinforced compound (5*A*…*x*+, 5*B*…*y*+), in addition to 12 nonreinforced test presentations of *w* or *z*, 6 preceded by *A* and 6 by *B*, along with 8 unsignalled presentations of *w* or *z* alone, 4 of which were reinforced (e.g., 4*w*+, 4*w*−, 6*A*…*w*−, 6*B*…*w*−); *w* and *z* sessions occurred in the same order as in the first test. The reinforced presentations of *w* and *z* were included to ensure the animals did not stop responding altogether on the test trials; as the critical comparison was between responding on same and different trials, rather than with responding to the target alone, this difference in reinforcement experience did not affect interpretation of the results.

###### 2.1.3.4.2 Test 3

Sessions 57–60 comprised only test trials; thus there were six reinforced presentations of *w*, six of *z*, and 15 nonreinforced presentations of each transfer CS signalled by each occasion setter (15*A*…*w*−, 15*A*…*z*−, 15*B*…*w*−, 15*B*…*z*−, 6*w*+−, 6*z*+).

#### 2.1.4 Data treatment

Responding during each stimulus type was assessed from the total number of responses for each trial type for each session (in responses per minute, rpm). During the test sessions the scores reported refer to the rate of responding during the transfer CS. A significance level of *p* < .05 was adopted. Data were analysed using factorial analysis of variance; significant interactions were examined with simple main effects analysis using the non-pooled error term (Howell, 2002, p. 490).

### 2.2 Results

#### 2.2.1 Positive patterning training

**Fig. 1:**
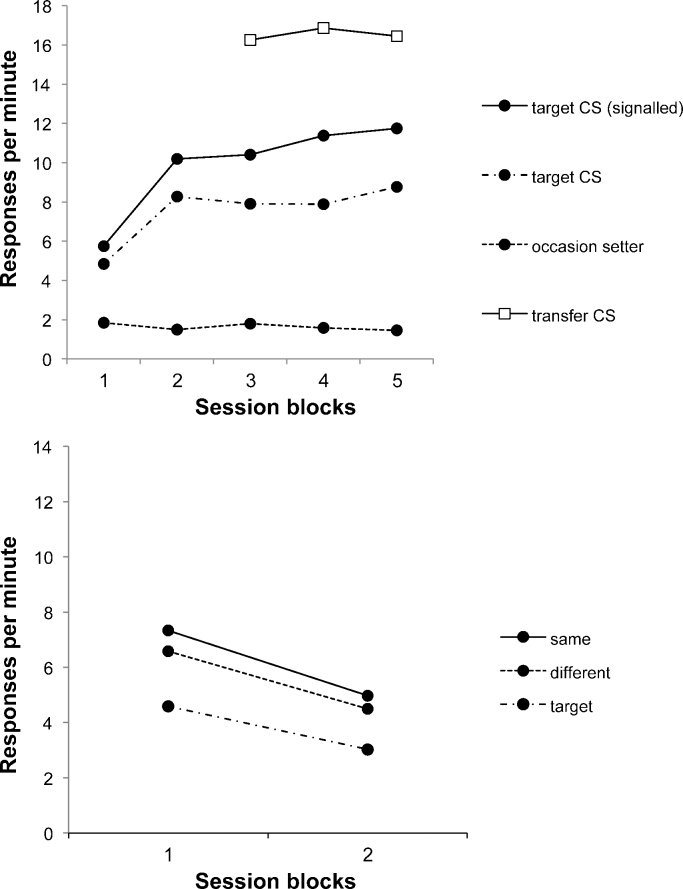
To*p panel*: Responding during stages 1 and 2 of Experiment 1, during the target CSs when signalled by the occasion setters and when presented alone; responding to the occasion setters and transfer CSs is also presented. Blocks 1–2 represent 8-session blocks, and blocks 3–5 6-session blocks. *Bottom panel*: Responding on same and different trials to the transfer CSs signalled by the occasion setters, and the transfer CSs alone, in the third test of Experiment 1. The data are presented in two-session blocks.

Responding to the target CS when it was signalled by the occasion setter and when it was presented alone is shown in the top panel of Fig. 1; the data are pooled into 8-session blocks for stage 1 and 6-session blocks for stage 2. ANOVA with block and trial type (target CS reinforced or nonreinforced) as factors revealed a significant interaction between these two factors, *F*(4,60) = 4.76, *p* = .002; the discrimination was significant on every block, smallest *F*(1,15) = 6.69, *p* = .02. Responding to the occasion setter remained low throughout these sessions. Responding to the transfer CSs was initially high, but fell during the final two sessions in which they were nonreinforced (not evident in Fig. 1 as the data are presented in 6-session blocks – see above); in the final session the mean response rate to these stimuli was 10.0 rpm.

#### 2.2.2 Test: occasion setters on transfer CSs

In each test responding to the transfer CSs was compared on *same* trials – on which the outcome previously signalled by occasion setter and transfer CS was the same – with that on *different* trials, on which it was not.

##### 2.2.2.1 Tests 1 and 2

The data from each of Tests 1 and 2 were calculated in two, 4-session blocks. In Test 1 the rate of responding on same trials was 10.65 and 10.06 rpm, and on different trials 10.28 and 9.72 rpm, for blocks 1 and 2, respectively; the corresponding means for Test 2 were 7.47 and 7.30, and 7.45 and 7.27 rpm. In neither test was there any difference between responding on same and different trials; ANOVAs with trial type (same/different) and blocks as factors revealed nothing significant, *F*'s < 1. The rates of pre-CS responding remained low for blocks 1 and 2, respectively, being .37 and .39 rpm for Test 1, and .35 and .38 rpm for Test 2.

##### 2.2.2.2 Test 3

The data from Test 3 are shown in the bottom panel of Fig. 1, in 2-session blocks. Here responding to the transfer CS was higher on same than on different trials: ANOVA with block and trial type (same or different) as factors revealed a significant effect of trial type, *F*(1,15) = 6.47, *p* = .023; the effect of block was also significant, *F*(1,15) = 17.12, *p* = .0009, but the interaction was not, *F* < 1. The rates of responding to the transfer CS alone were 4.59 and 3.03 rpm in blocks 1 and 2, respectively, and this was lower than responding on compound trials, *F*(1,15) = 15.90, *p* = .001. The rates of pre-CS responding remained low, at .37 and .24 rpm in blocks 1 and 2, respectively.

### 2.3 Discussion

An occasion setter that had signalled reinforcement of a target CS with a particular outcome was more effective at promoting responding to a transfer CS paired with the same outcome than to one paired with a different outcome. This finding supports the prediction made by the hierarchical account, according to which the occasion setter operates on a specific CS–US association. A similar result was reported by Morell and Davidson (2002), although in their study, in contrast to our own, exposure to the outcomes presented on same and different trials was not equated.

**Fig. 2:**
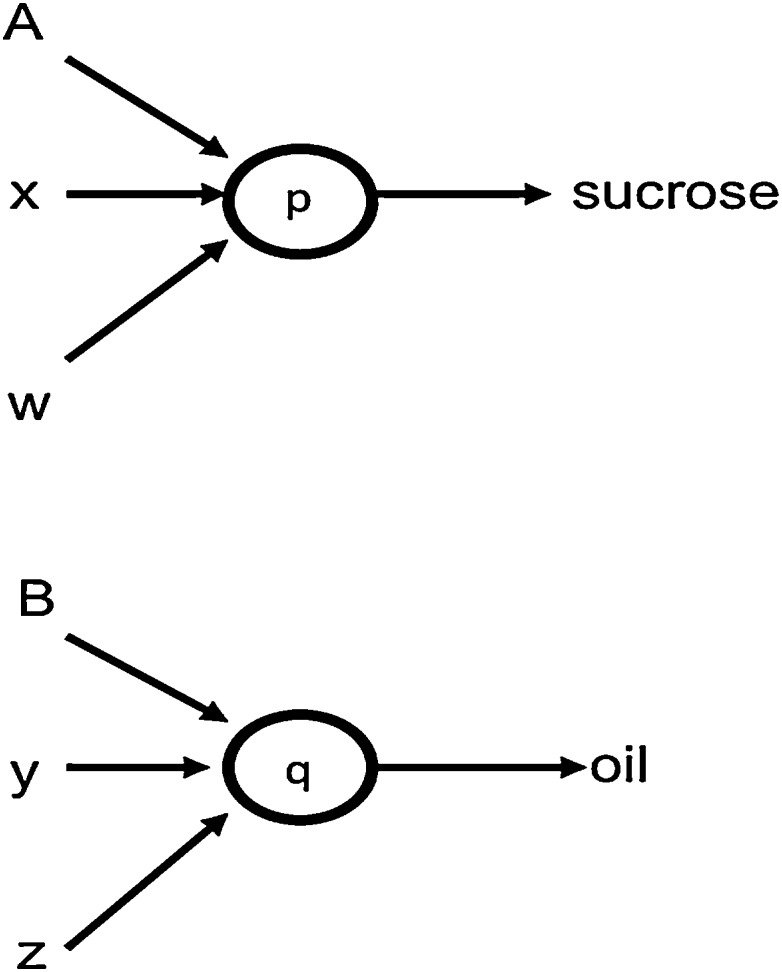
Representation of the associative structure proposed by Honey and Watt (1998) to result from training in Experiment 1, in which *A* signals reinforcement of *x* by sucrose, and *B* reinforcement of *y* by oil; *w* and *z* are independently paired with sucrose and oil respectively.

Standard configural theory cannot explain these findings, because it predicts that test responding depends on the similarity between the configural cue that was conditioned and that present at test – and these configural cues do not contain a representation of the outcome (cf. Honey and Watt, 1999). It follows that an adaptation of configural theory that allowed the outcomes to be represented in the configural cues *could* explain these results. For example, training *A* so that it signalled reinforcement of *x* with sucrose would have resulted in conditioning to the configural cue *A*/*suc*/*x*/*suc*. If *w* is subsequently paired with sucrose and *z* with oil, when *A* signals *w* and *z* at test, the resulting configural cues will be *A*/*suc*/*w*/*suc* and *A*/*suc*/*z*/*oil* – and as the former is more similar to the training configure than the latter, this will result in more responding in the former case. An example of such a pseudo-configural theory was proposed by Honey and Watt (1999), who argued that if *A* signals reinforcement of *x* with sucrose, *A* and *x* become linked to a common hidden unit, *p*, that also becomes linked to the representation of sucrose. When *w* is subsequently paired with sucrose, activity in the sucrose representation feeds back to *p*, and allows *w* to become associated with it as well (see Fig. 2). A similar associative structure would link *B*, *y* and *z* to the oil representation via a second hidden unit, *q*. Assuming that two sources of activation to one hidden unit produce more activation than one source of activation to two hidden units (cf. Honey and Watt, 1998, p. 333), then the test results can be explained, as *A* and *w* both activate the same hidden unit, *p*, whereas *A* and *z* do not.

Although arguably not a true configural theory in terms of the definition given above, Honey and Watt's suggestion shares with configural theories the implicit assumption that occasion setters and target CSs play qualitatively identical roles in discrimination performance, so that replacing either should degrade performance in a qualitatively equivalent manner. This contrasts with the hierarchical view, according to which there is an inherent functional asymmetry between *A* and the target *x* on which it operates – while *x* simply activates the US representation, *A* expedites this process, by facilitating the flow of activation between *x* and sucrose (Holland, 1983). The distinction between these rival classes of account motivated the experiments that follow.

## 3 Experiment 2

In Experiment 2 animals were again trained on two positive patterning discriminations, in which *A* and *x* signalled sucrose and *B* and *y* signalled oil, and trained with two transfer CSs, *w* and *z*, the former being paired with sucrose and the latter with oil. They were also trained with two *pseudo-occasion setters*, *C* and *D*, which were also visual cues reinforced 15 s after their offset, so that they had the same temporal relation with reinforcement as the true occasion setters; one was paired with sucrose and the other with oil (see Table 2). Again the animals were tested by replacing a component of the reinforced compound from the occasion-setting discrimination with a transfer stimulus. However, rather than replacing the target CS *x* with one of the transfer CSs *w* or *z*, the occasion setter *A* was replaced with one of the pseudo-occasion setters *C* or *D*. The pseudo-configural theory proposed by Honey and Watt (1999) assumes that occasion setter and target both contribute in a qualitatively similar way to the common hidden unit that commands responding, albeit to greater or lesser extents, and so would predict more responding on same than on different trials – the same result observed in the final test of Experiment 1. But because the essence of hierarchical theory is that occasion setters and CSs have qualitatively different properties, replacing the occasion setter would remove that element of the task that made it hierarchical, and so the theory would *not* be constrained to make this prediction.

### 3.1 Method

#### 3.1.1 Subjects

The subjects were 16 male hooded Lister rats with a mean *ad lib.* weight of 277 g (range = 250–290 g). They were deprived and maintained as in the previous experiment.

#### 3.1.2 Apparatus

This was the same as in the previous experiment, except that two additional visual stimuli were employed: dark was achieved by turning off the houselight, and a flash by alternating .40 s presentations of a 2.8-W bulb situated in the centre of the ceiling with .40 s presentations of the dim houselight, these being separated by .20 s of darkness.

#### 3.1.3 Procedure

All details not specified in this and the following experiments were identical to those of Experiment 1.

##### 3.1.3.1 Pretraining

Subjects were first magazine trained to eat sucrose and oil.

##### 3.1.3.2 Stage 1: positive patterning training

Subjects then received 18 sessions of positive patterning.

##### 3.1.3.3 Stage 2: transfer CS training

In sessions 19–38 animals were given further positive patterning training sessions, and were also conditioned to the four transfer stimuli. For all animals *C* and *w* were paired with sucrose (the flash and buzz, respectively), and *D* and *z* (dark and tone, respectively) were paired with oil; however, whereas *w* and *z* were followed immediately by their respective reinforcers, *C* and *D* were reinforced after a 15 s trace interval, to match the temporal relations between A and B and reinforcement. These sessions were identical to sessions 1–18 except that there were 12 of each of the four types of nonreinforced trial, 6 of each reinforced compound trial, and 3 reinforced trials with each of the four transfer stimuli, yielding a total of 72 trials per session (12*A*−, 12*B*−, 12*x*−, 12*y*−, 6*A*…*x*+, 6B…*y*+, 3*w*+, 3*z*+, 3*C*…+, 3*D*…+). In a further 4-session block of training *w* and *z* were extinguished (sessions 39–42). (We did not also extinguish *C* and *D* because rates of responding to these stimuli were already relatively low, and any further reduction would introduce the possibility that we would obtain a null effect at test through a floor effect.^2^)

##### 3.1.3.4 Test: pseudo-occasion setters on target CSs

The test sessions 43–46 were identical to those of the final test of Experiment 1, except that *C* and *D* substituted for *A* and *B*, and *x* and *y* for *w* and *z* – so that the pseudo-occasion setters *C* and *D* signalled the target CSs *x* and *y*. When *x* and *y* were presented alone they were followed by their respective reinforcers, sucrose and oil. This was both to maintain comparability with the occasion setter/transfer CS test of Experiment 1, and also to maintain delivery of reinforcement in the test sessions, without which the animals would have been likely to stop responding altogether.

#### 3.1.4 Data treatment

The data from positive-patterning training were presented in 6-session blocks for Stage 1, and 8-session blocks for Stage 2. During the test sessions the scores reported refer to the rate of responding during the target CS.

### 3.2 Results

#### 3.2.1 Positive patterning training

**Fig. 3:**
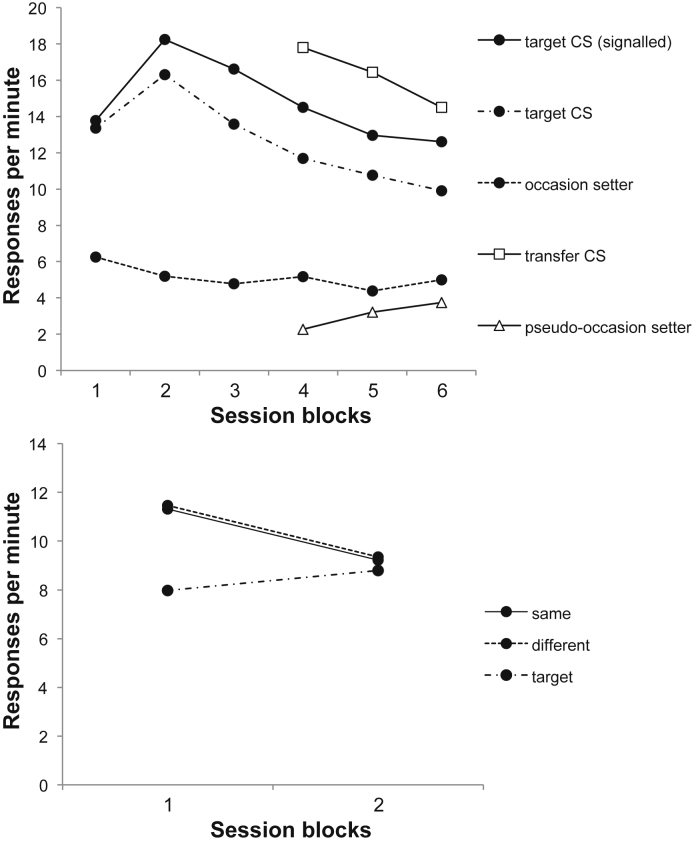
To*p panel* Response rates during stages 1 and 2 of Experiment 2, during the target CSs alone and when signalled by the occasion setters, the occasion setters alone, the transfer CSs, and the pseudo-occasion setters are presented. Blocks 1–3 represent 6-session blocks, and blocks 3–6 8-session blocks. *Bottom panel*: Response rates on same and different trials during the target CSs signalled by the pseudo-occasion setters, and the target CSs alone, in the test of Experiment 2. The data are presented in two-session blocks.

ANOVA with session block (1–6) and trial type (target CS reinforced or nonreinforced) as factors revealed main effects of block and of trial type, *F*(5,75) = 4.17, *p* = .002 and *F*(1,15) = 13.18, *p* = .003; the interaction was not significant, *F*(5,75) = 1.53, *p* = .19; this confirmed that the animals had learned the discrimination (Fig. 3, top panel). There was little response to the occasion setter alone during these sessions, but the animals responded at a high rate to the transfer CSs, *w* and *z*, and slightly less to the pseudo-occasion setters. In the final training session responding to *w* and *z* was 8.91 rpm, to *C* and *D* 4.75 rpm, and in the trace interval 5 s after their offset (the interval during which the target CS would be presented at test) 10.80 rpm.

#### 3.2.2 Test: pseudo-occasion setters on target CSs

The lower panel of Fig. 3 suggests that there was little sign of a difference in responding on same and different trials. ANOVA with trial type (same, different) and block as factors revealed only a main effect of blocks, *F*(1,15) = 4.96, *p* = .04; the effect of trial type and the interaction were not significant, *F*'s < 1. The mean rates of target responding were 7.97 and 8.80 rpm for blocks 1 and 2, respectively; these differed from responding on the test trials on block 1 *F*(1,15) = 21.94, *p* < .001. The rates of pre-CS responding were 1.57 and 1.14 rpm for blocks 1 and 2, respectively.

### 3.3 Discussion

After training on two feature-positive discriminations, animals in Experiment 1 received a test in which the target CS was replaced by a transfer CS, and those in Experiment 2 a test in which the occasion setter was replaced by a pseudo-occasion setter. In the former they responded more on same than on different trials, in the latter they did not. These results are not consistent with pseudo-configural theory, which assumes that occasion setter and target CS play functionally equivalent roles in generating responding, so that replacing one should have the same effect as replacing the other. However, even though a difference was observed at test in Experiment 1 but not in Experiment 2, there were differences between the two studies that limit the reliability of this cross-experiment comparison. For example, in Experiment 2 both transfer CSs and pseudo-occasion setters were trained, whereas in Experiment 1 they were not. Thus one aim of Experiment 3 was to replicate both findings under comparable training conditions. Second, although the same/different comparisons within each test were perfectly counterbalanced, such that the physical identity of the same and different test trial combinations were identical, different stimulus sets were used for the two types of test. More specifically, the effect of jewel and traylight occasion setters on buzz and tone transfer CSs was examined in the test of Experiment 1, and the effect of flash and dark pseudo-occasion setters on click and noise target CSs in the test of Experiment 2. If the stimuli used for the pseudo-occasion setter test were less discriminable from each other, this could explain the pattern of results observed. Therefore, in Experiment 3 the stimulus sets used for the two test types were reversed, so that the effect of flash and dark occasion setters on click and noise transfer CSs, and jewel and traylight pseudo-occasion setters on buzz and tone target CSs was examined. If an advantage of responding on same trials is obtained in the occasion setter but not the pseudo-occasion setter test regardless of which stimulus sets serve in the two types of test, then the results cannot be attributed to differences in stimulus discriminability.

## 4 Experiment 3

Experiment 3 was conducted in two replications (see Table 3). In both, animals were given identical training to that of Experiment 2, except that the stimulus sets used for occasion-setting training and for the transfer stimuli in that study were reversed (i.e. *A*, *B*, *x* and *y* were dark, flash, buzz and tone, and *C*, *D*, *w* and *z* were jewels, tray, click and noise). In Experiment 3a animals were tested with the original occasion setters and the transfer CSs (*A*…*w*, *A*…*z*, *B*…*w*, *B*…*z*) as in Experiment 1 (see Table 1), and in Experiment 3b with the pseudo-occasion setters and the target CSs (*C*…*x*, *C*…*y*, *D*…*x*, *D*…*y*) as in Experiment 2. This ensured that the test compounds used for the occasion setter test in Experiment 1 were used for the pseudo-occasion setter test here, and those used for the pseudo-occasion setter test in Experiment 2 were used for the occasion setter test here.

### 4.1 Method

#### 4.1.1 Subjects

The subjects in Experiment 3a were 16 male hooded Lister rats with a mean *ad lib.* weight of 287 g (range = 270–315 g); the 16 subjects in Experiment 3b had a mean weight of 296 g (range 280–310). They were deprived and maintained as in the previous experiment.

#### 4.1.2 Apparatus

As Experiment 1.

#### 4.1.3 Procedure

##### 4.1.3.1 Pretraining

As Experiment 1.

##### 4.1.3.2 Stage 1: positive patterning training

As Experiment 2, except that flash and dark served as the occasion setters *A* and *B*, and buzz and tone as the target CSs *x* and *y*. Thus for half the animals the flash signalled reinforcement of the buzz, and dark reinforcement of the tone; for the remaining subjects this arrangement was reversed. For half of each subgroup the buzz signalled sucrose and the tone oil, and for the remainder the reverse.

##### 4.1.3.3 Stage 2: transfer CS training

As Experiment 2, except that noise and the click served as *w* and *z* respectively, and jewels and tray light as *C* and *D* respectively; as in Experiment 2, *C* and *w* were paired with sucrose, and *D* and *z* with oil. In addition, in Experiment 3a, despite employing identical training procedures to all previous studies, rats stopped consuming all their oil during the training sessions. In an attempt to address this, in sessions 21–24 the number of reinforced trials per session was reduced (for sessions 21–22: 13*A*−, 13*B*−, 13*x*−, 13*y*−, 4*A*…*x*+, 4*B*…*y*+, 3*w*+, 3*z*+, 3*C*…+, 3*D*…+, and for sessions 23–24: 15*A*−, 15*B*−, 15*x*−, 15*y*−, 2*A*…*x*+, 2*B*…*y*+, 2*w*+, 2*z*+, 2*C*…+, 2*D*…+). However, the problem persisted, so from session 25 onwards we reverted to the trial numbers employed in sessions 19 and 20, but reduced the volume of oil per delivery to .18 ml for the rest of this phase (by setting the pumps for 100 cs rather than 160 cs), which proved effective. Thus the number of each type of compound, nonreinforced target/occasion setter and reinforced transfer CS trial was 240, 566 and 58 trials respectively in Experiment 3a, and 252, 558 and 60 in Experiment 3b.

##### 4.1.3.4 Test

In the test of Experiment 3a the original occasion setters *A* and *B* signalled the transfer CSs *w* and *z* (exactly as in Test 3 of Experiment 1), while in Experiment 3b the pseudo-occasion setters *C* and *D* signalled the original target CSs *x* and *y* (exactly as in the test of Experiment 2).

##### 4.1.3.5 Data treatment

During the test sessions the scores reported refer to the rate of responding during the transfer CS in Experiment 3a, and the target CS in Experiment 3b.

### 4.2 Results

#### 4.2.1 Positive patterning training

**Fig. 4:**
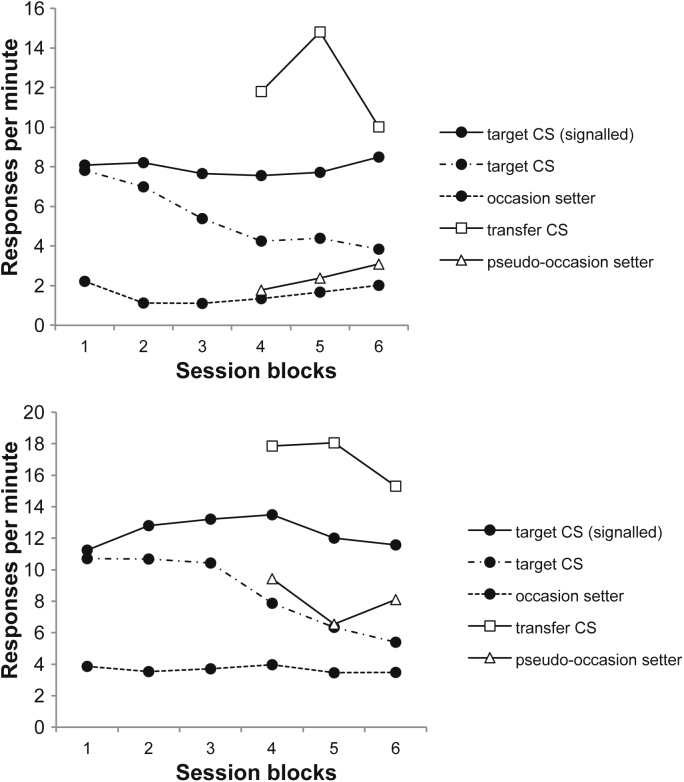
Response rates during stages 1 and 2 of Experiment 3a (top panel) and Experiment 3b (bottom panel), during the target CSs alone and when signalled by the occasion setters, the occasion setters alone, the transfer CSs, and the pseudo-occasion setters are presented. Blocks 1–3 represent six-session blocks, and blocks 3–6 eight-session blocks.

Discrimination performance from Experiments 3a and 3b may be seen in the upper and lower panels of Fig. 4 respectively.

##### 4.2.1.1 Experiment 3a

ANOVA with session block (1–6) and trial type (target CS reinforced versus nonreinforced) as factors revealed a interaction between these two factors, *F*(5,75) = 8.10, *p* < .001; the discrimination was significant in blocks 2–6, smallest *F*(1,15) = 11.12, *p* = .005. In the last training session the mean response rate to the pseudo-occasion setters was 3.60 rpm and in the interval 5 s after their offset during which the target CS would be presented at test, 6.13 rpm, while that to the transfer CSs was 8.75 rpm.

##### 4.2.1.2 Experiment 3b

ANOVA performed on the corresponding data from Experiment 3b also revealed a significant interaction, *F*(5,75) = 18.54, *p* < .001; the discrimination was significant on blocks 2–6, smallest *F*(1,15) = 10.29, *p* = .006. In the last training session the mean response rate to the pseudo-occasion setters was 5.00 rpm and in the trace interval 5 s after their offset 5.31 rpm, and that to the transfer CSs 10.19 rpm.

#### 4.2.2 Test

**Fig. 5:**
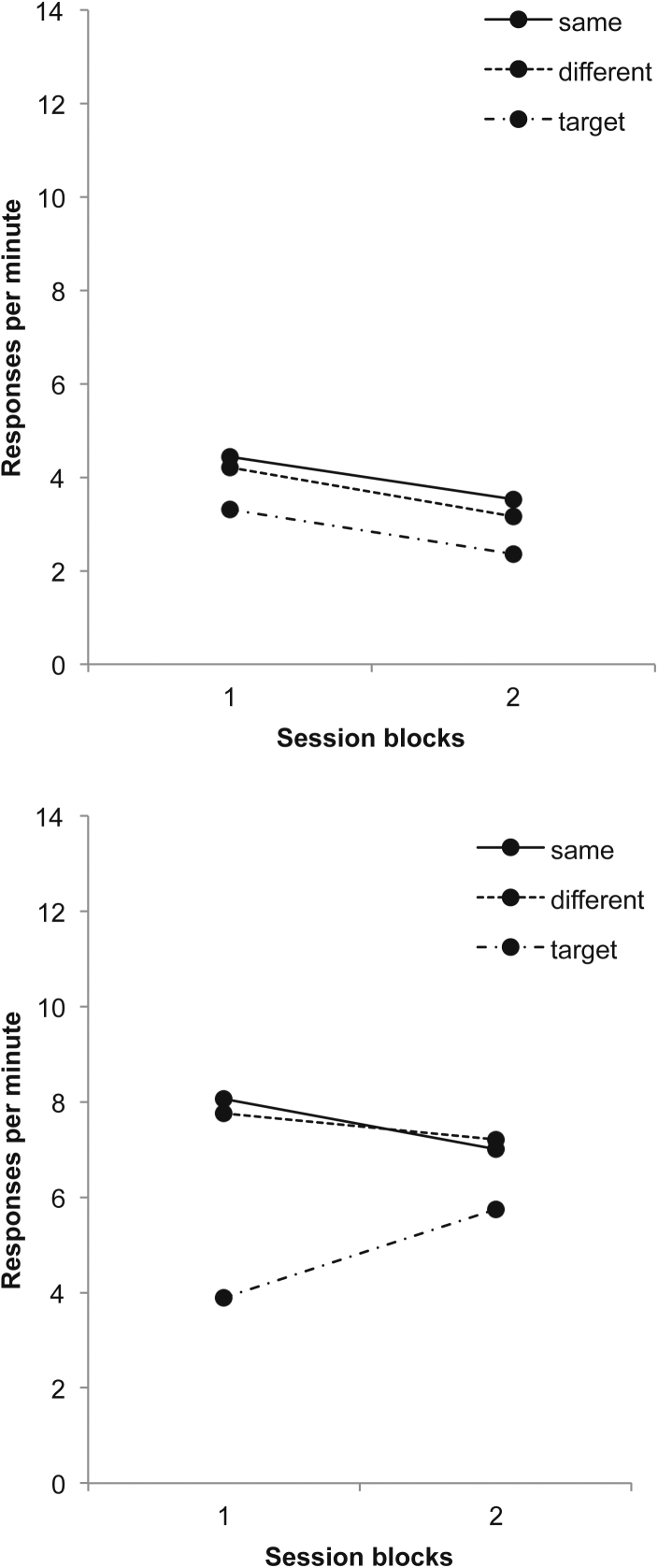
*Top panel:* Response rates on same and different trials during the transfer CSs signalled by the occasion setters in Experiment 3a; responding to the transfer CSs alone is also depicted. *Bottom panel*: Responding to the target CSs signalled by the pseudo-occasion setters in Experiment 3b; responding to the target CSs alone is also depicted. The data are presented in two-session blocks.

The test data for Experiments 3a and 3b are shown in the top and bottom panels of Fig. 5 respectively.

##### 4.2.2.1 Experiment 3a: occasion setters on transfer CSs

As in Experiment 1, responding to the transfer CSs was greater on same trials than on different trials (top panel of Fig. 5). ANOVA with trial type (same, different) and blocks as factors revealed a main effect of trial type, *F*(1,15) = 5.48, *p* = .034; the main effect of blocks was also significant, *F*(1,15) = 10.56, *p* = .005, but the interaction was not, *F* < 1. The mean rate of responding to the target CSs alone was 3.31 rpm in block 1 and 2.36 rpm in block 2, and these rates differed from test trial responding, *F*(1,15) = 8.56, *p* = .01. The rates of pre-CS responding were .61 and .58 rpm for blocks 1 and 2 respectively.

##### 4.2.2.2 Experiment 3b: pseudo-occasion setters on target CSs

As in Experiment 2, there was little sign of a consistent difference in responding on same and different trials to the target CSs when signalled by the pseudo-occasion setters (bottom panel of Fig. 5); ANOVA with trial type (same, different) and blocks as factors revealed no effect of trial type, *F* < 1, and no significant effects or interactions, largest *F*(1,15) = 2.70, *p* = .12 for the effect of block; the interaction was not significant, *F*(1,15) = 1.07, *p* = .32. In order to investigate whether the slight advantage in responding on same trials in the first block was reliable, a further ANOVA was conducted on this block, but this also was nonsignificant, *F* < 1. The mean rate of responding to the target CSs was 3.89 rpm in block 1 and 5.75 rpm in block 2; this was lower than test trial responding on block 2, *F*(1,15) = 8.94, *p* = .001. The rates of pre-CS responding were 1.02 and 1.39 rpm for blocks 1 and 2 respectively.

##### 4.2.2.3 Experiment 3a and 3b

To compare performance in the two experiments directly, a ratio of same/different responding was calculated for each block in each test; 1.0 represented indifference between the two types of trial, whereas ratios of greater than 1.0 reflect higher responding on same than on different trials (the use of ratios was intended to compensate for the added variance introduced by differing levels of responding in the two tests). The resultant ratios were 1.25 and 1.67 for Experiment 3a, and 1.05 and 1.01 for Experiment 3b. ANOVA with Experiment (3a and 3b) and block as factors revealed a main effect of experiment, *F*(1,30) = 5.49, *p* = .027 – confirming that the tendency to respond more on same than on different trials was significantly greater in Experiment 3a; nothing else was significant, *F*s < 1. As the interaction with block was not significant, the two ratios were averaged for each rat, and a one sample *t*-test established that the ratio differed from 1 in Experiment 3a, *p* = .02, but not in Experiment 3b, *p* = .54. This confirmed that responding was higher on same than on different trials in Experiment 3a, when the occasion setters were tested with the transfer CSs, but not in Experiment 3b, where the pseudo-occasion setters were tested with the target CSs.

### 4.3 Discussion

The results of Experiment 3 confirm those of Experiments 1 and 2; transfer of the occasion setters to the transfer CSs was US-specific, whereas transfer of the pseudo-occasion setters to the target CSs was not – results which are inconsistent with the predictions of pseudo-configural theory. Nonetheless, although the predictions of pseudo-configural theory must be qualitatively the same for the two kinds of tests, they are not constrained to be quantitatively the same; for example, this account could assert that the occasion setters, being less temporally adjacent to the US than the target CS, contributed correspondingly less to the “configural” hidden units. This would mean that substituting the occasion setter with another stimulus would have less effect on responding than substituting the target CS, and could explain why US specificity was only observed in the latter case.

This asymmetry in training of occasion setters and target CSs was inevitable, as our intention was to bias the task towards one that required a hierarchical solution. If both stimuli had been trained simultaneously in an identical temporal relation to the reinforcer, then the animal would have no way of identifying one as the occasion setter and the other as the target. This would make a hierarchical interpretation unhelpful, and force the animal to resort to a configural solution (see Holland, 1989 for evidence that serial presentation fosters a hierarchical solution to discriminations of this type). The possibility that pseudo-configural theory could underlie the effects we observed therefore needs to be ruled out in some other way.

As outlined above, Honey and Watt's (1999) pseudo-configural account can explain greater responding on same than on different trials by assuming that two sources of activation to one hidden unit produce more activation than one source of activation to two such units. Without extra assumptions, such an account must therefore always predict more responding on same than on different trials regardless of whether the occasion setter is present or not – although it can explain *lack* of an effect in terms of reduced sensitivity. It can, however, never predict the opposite result – *less* responding on same than on different trials. Hierarchical theory is not constrained in this manner. This is because, according to this account, the same-trial advantage in the occasion setter/transfer CS test occurs because of the way in which occasion setters control responding to target CSs – so if no occasion setters are present, it makes no special prediction. In this case the outcome would depend solely on the principles of summation of Pavlovian CSs.

But what would Pavlovian summation be predicted to yield in this task? Some prior studies have examined whether Pavlovian summation is greater when the two compounded CSs signal the same or different outcomes, but their findings are ostensibly inconsistent with each other. For example, Ganesan and Pearce (1988) used a between-subjects design, with food and water as the two outcomes, and found no difference at test in responding to compounds comprising stimuli paired with either the same or different outcomes. However, the groups given these same and different tests also differed in their experience with the two outcomes, which is a potentially confounding factor. Two further studies have employed within-subjects designs, and avoided this problem. Rescorla (1999) reported a study using food and liquid sucrose reinforcers, in which he found more responding to a compound of cues that had signalled the same outcome during training. In contrast Watt and Honey (1997) conducted a formally similar study with the same reinforcers, but reported the opposite result – more responding to the compound of cues that had signalled different outcomes. However, this apparent contradiction can perhaps be attributed to methodological differences. One very salient procedural difference between the two studies was in the presentation of the test compounds – in Rescorla's study the cues were presented simultaneously, whereas in Watt and Honey's experiment the cues were presented serially, with one stimulus immediately following presentation of the other – an arrangement which is much more similar to that used here. Thus, based on the similarity of our procedures to those employed by Watt and Honey, we might predict that Pavlovian summation should produce more responding on different trials in the present experiment.

## 5 Experiment 4

The preceding argument allows us to experimentally discriminate between the predictions of the hierarchical account and pseudo-configural theory. Pseudo-configural theory explains the same trial advantage we observed in Experiments 1–3 in terms of dual activation of one US representation being superior to single activation of two US representations. Thus this same-trial advantage should always be observed provided the CSs are strongly enough associated with their outcomes – the opposite pattern should never occur. Hierarchical theory, in contrast, envisages two independent processes. The same trial advantage, according to this account, is only observed if an occasion setter is present, because it is produced by selective transfer of the occasion setter to target CSs that signal the original outcome. If the occasion setter is not present, then Pavlovian processes will predominate – which in this procedure would produce the opposite pattern, more responding on different trials. This is the prediction that was tested in the present experiment.

After the same training as in Experiments 2 and 3, different combinations of the trained cues were tested in compound – but never the stimuli that had served as occasion setters (see Table 4). In Test 1 the effect of the pseudo-occasion setters on responding to the target CSs was again examined; Test 2 evaluated the effect of the pseudo-occasion setters on responding to the *transfer* CSs and Test 3 the effect of the target CSs on responding to the transfer CSs. This allowed us to investigate the pattern of Pavlovian summation in this task. The pseudo-configural theory predicts that the same-trial advantage will persist in these various tests, or that no difference will observed. However, in contrast to the hierarchical account, the pseudo-configural theory cannot accommodate the opposite result, greater responding on different trials.

### 5.1 Method

#### 5.1.1 Subjects

The subjects were 16 male hooded Lister rats with a mean *ad lib.* weight of 338 g (range = 320–380 g).

#### 5.1.2 Apparatus

As Experiment 1.

#### 5.1.3 Procedure

##### 5.1.3.1 Pretraining

As Experiment 1.

##### 5.1.3.2 Stage 1: positive patterning training

As in Experiment 2.

##### 5.1.3.3 Stage 2: transfer CS training

As Experiment 2, except that the dark and flash were counterbalanced across the two reinforcer types, as were the buzz and the tone. This was necessary to ensure that the stimulus compounds comprising same and different trials in the test of the pseudo-occasion setters on the transfer targets were matched in stimulus identity. Thus for half the animals the jewel lights signalled reinforcement of the click, and the traylight reinforcement of the noise; for the remaining subjects this arrangement was reversed. For half of each subgroup the click signalled sucrose and the noise oil, and for the remainder the reverse. For half of each of these four subgroups the buzz was paired with sucrose and the tone with oil, and for the remainder the reverse; for half of these eight subgroups the flash was paired with sucrose and the dark with oil, and for the remainder the reverse.

##### 5.1.3.4 Test

###### 5.1.3.4.1 Test 1: pseudo-occasion setters on target CSs

Identical to the test of Experiment 2.

###### 5.1.3.4.2 Test 2: pseudo-occasion setters on transfer CSs

After four retraining sessions identical to those of the transfer CS training stage, in which *w* and *z* were reinforced, the animals received Test 2 which was identical to Test 1, except that *w* and *z* were substituted for *x* and *y*. In sessions 1 and 4*w* and *z* were reinforced when presented alone, and in sessions 2 and 3 *C* and *D*.

###### 5.1.3.4.3 Test 3: transfer CSs on target CSs

Four more sessions of retraining were followed by Test 3, which was identical to Test 1 except that the pseudo-occasion setters were replaced by the transfer CSs, so that the effect of *w* and *z* on responding to *x* and *y* could be evaluated.

###### 5.1.3.4.4 Data treatment

During the test sessions the scores reported refer to the rate of responding during the target CS in Tests 1 and 3, the transfer CS in Test 2.

### 5.2 Results

#### 5.2.1 Positive patterning discrimination

**Fig. 6:**
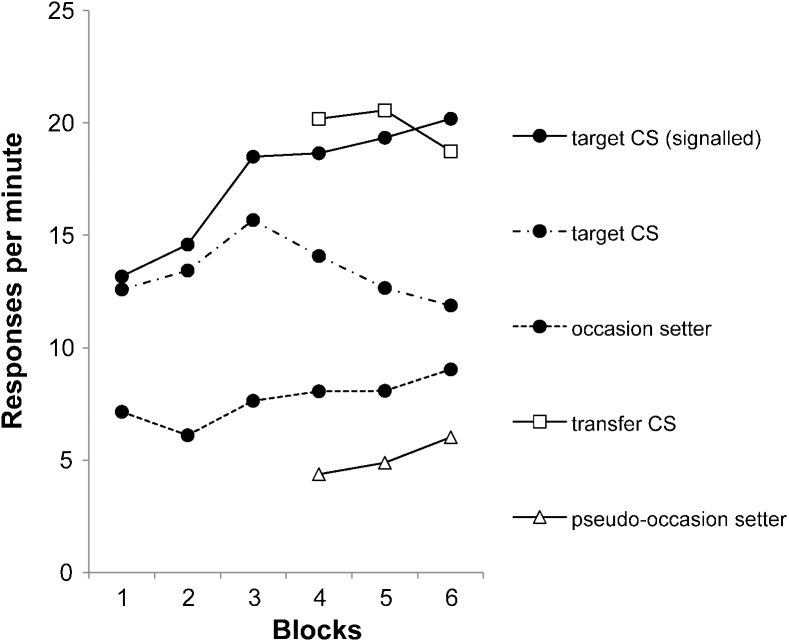
Response rates during stages 1 and 2 of Experiment 4, to the target CSs alone and when signalled by the occasion setters, the occasion setters alone, the transfer CSs, and the pseudo-occasion setters. Blocks 1–3 represent 6-session blocks, and blocks 3–6 8-session blocks.

Performance on the positive patterning discrimination may be seen in Fig. 6. ANOVA with session block (1–6) and trial type (target reinforced or not) as factors revealed a significant interaction between these two factors, *F*(5,75) = 16.96, *p* < .001; the discrimination was significant on blocks 2–6, smallest *F*(1,15) = 7.02, *p* = .02. In the last training session the mean response rate to the pseudo-occasion setters was 5.63 rpm and in the interval 5 s after their offset 11.25 rpm; that to the transfer CSs was 10.88 rpm.

#### 5.2.2 Test 1: pseudo-occasion setters on target CSs

**Fig. 7:**
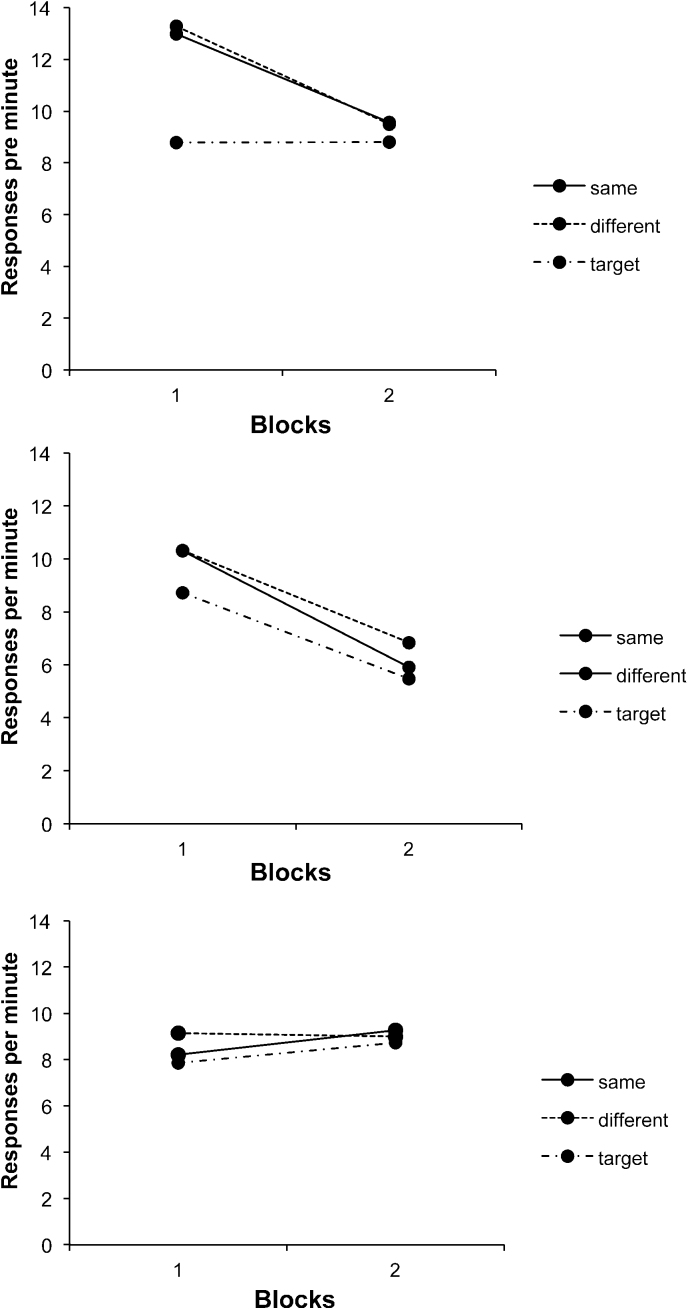
Response rates on same and different trials during the three tests of Experiment 4; data are presented in two-session blocks. *Top panel*: Pseudo-occasion setters signal target CSs; responding to the target CSs alone is also depicted. *Centre panel*: Pseudo-occasion setters signal transfer CSs; responding to the transfer CSs alone is also depicted. *Lower panel:* Transfer CSs signal target CSs; responding to the target CSs alone is also depicted.

Once more there was no sign of a difference in responding on *same* and *different* trials (Fig. 7 top panel); ANOVA with block and trial type as factors revealed a significant effect of block, *F*(1,15) = 22.03, *p* = .0003; nothing else was significant, *F*s < 1. Responding on target alone trials was 8.78 and 8.80 rpm for blocks 1 and 2; these rates differed from test trial responding on block 1, *F*(1,15) = 32.63, *p* < .001. The rates of pre-CS responding were 1.71 and 1.25 rpm for blocks 1 and 2 respectively.

#### 5.2.3 Test 2: pseudo-occasion setters on transfer CSs

By the second block of the second test there appeared to be slightly superior responding on different trials (centre panel of Fig. 7); however, ANOVA with block and trial type as factors revealed only an effect of block, *F*(1,15) = 29.04, *p* = .0001; there was no effect of trial type, *F*(1,15) = 2.03, *p* = .17, and the interaction was nonsignificant, although marginal, *F*(1,15) = 3.08, *p* = .09;nonetheless, when analysed separately, the effect on block 2 was significant *F*(1,15) = 6.24, *p* = .025. Responding on target alone trials was 8.72 and 5.47 rpm for blocks 1 and 2; these rates differed from test trial responding, *F*(1,15) = 15.28, *p* = .001. The rates of pre-CS responding were 2.30 and 1.95 rpm for blocks 1 and 2 respectively.

#### 5.2.4 Test 3: transfer CSs on target CSs

Here responding on different trials again appeared greater on block 1 (bottom panel of Fig. 7). ANOVA with block and trial type revealed a significant interaction, *F*(1,15) = 4.64, *p* = .048, and responding on different trials was higher than that on same trials on block 1, *F*(1,15) = 6.34, *p* = .024. Responding on target alone trials was 7.86 and 8.72 rpm for blocks 1 and 2; these rates did not differ from responding to the target CSs alone, *F* < 1. The rates of pre-CS responding were 1.52 and 2.13 rpm for blocks 1 and 2 respectively.

### 5.3 Discussion

Three different combinations of cues were tested, none of which involved the occasion setters. There was no evidence of greater responding on same than on different trials in any of the tests, but there was evidence of the opposite–higher responding on different trials. Pseudo-configural theory cannot provide a ready explanation of this pattern of results. They are, however, consistent with a hierarchical interpretation. This account makes no specific prediction about the pattern of results that will be obtained if no occasion setter is present, but instead appeals to the principles of Pavlovian summation – which, on the basis of prior work (Watt and Honey, 1997), we have taken to predict more responding on different trials.

But why should summation of two Pavlovian CSs produce more responding on different trials? One possibility (cf. Watt and Honey, 1997) appeals to the idea that there is a ceiling to the degree to which a particular US representation can be activated (cf. Watt and Honey, 1997). For example, if a particular CS can activate the US representation to its optimal level, adding a second CS for the same outcome cannot produce a substantial increase in activation of that same representation. Adding a CS for a different outcome, in contrast, would produce a near maximal activation of the second US representation, and a correspondingly larger increase in conditioned responding. A second issue is why no sign of a parallel effect was ever observed in the test of the pseudo-occasion setters with the target CSs. This could perhaps be explained by generalisation: if there were any generalisation between the visual occasion setters and the visual pseudo-occasion setters, then presenting the pseudo-occasion setters with the target CSs might produce a small tendency for animals to respond more on same than on different trials, which would offset the Pavlovian summation-produced tendency for the opposite result. Such generalisation would be less likely when an auditory CSs preceded the target CSs in Test 3, or in Test 2 when the effect on the transfer CS was examined.

## 6 General discussion

These data add to an existing body of evidence that the configural class of theories cannot explain all aspects of occasion setting. For example, Holland (1989) has reported findings consistent with the idea that simultaneous patterning tasks selectively encourage configural learning, while serial patterning tasks foster an occasion-setting solution. He proposed that when training conditions favour perceptual discontinuity between occasion setter and target (for example, serial presentation, and when feature and target differ in modality), configuring is more difficult and a hierarchical strategy predominates (see also Bonardi and Jennings, 2007).

**Fig. 8:**
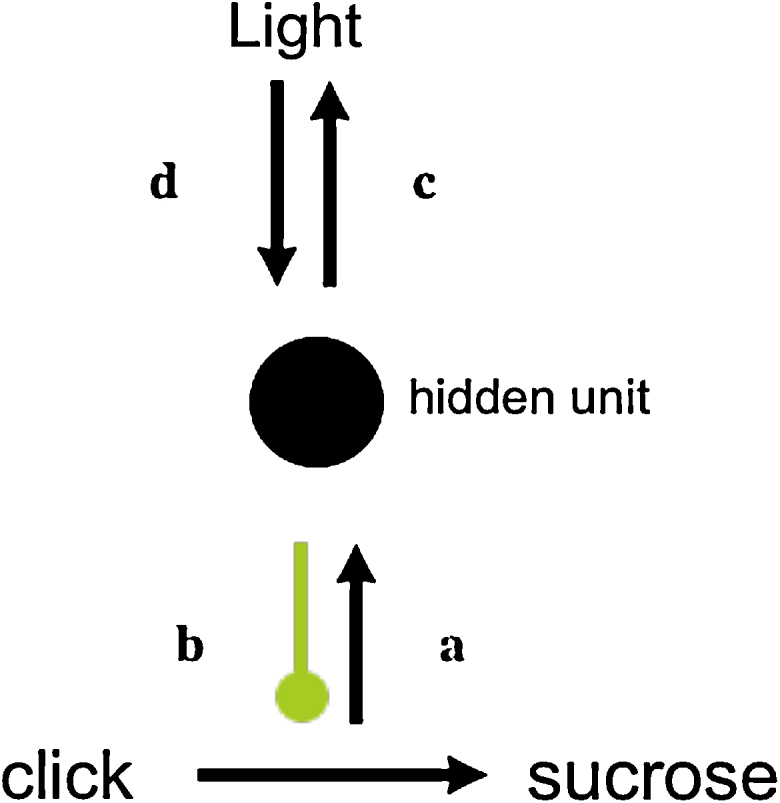
Representation of hierarchical structure forming when a light is an occasion setter for a clicker → sucrose association.

Nonetheless, in more recent years increasing emphasis has been placed on configural-type explanations of occasion setting effects, and there has been a corresponding tendency to neglect the possibility that hierarchical processes could explain performance on nonlinear tasks. As we have seen, one strong contender within the set of models aligned to the configural approach is the pseudo-configural account proposed by Honey and Watt (1998). Yet despite the many experiments whose findings support the predictions of this model (e.g., Close et al., 2009; Honey and Watt, 1998), virtually all of these results are equally consistent with an elaborated hierarchical account. More specifically (following an architecture proposed by Holland, 1992), one could assume that when a target CS → US association forms, a hierarchical architecture results (Fig. 8) that comprises the association itself (click → sucrose), a hidden unit that is specific to the click → sucrose association, a standard associative link *a* (depicted by an arrow) going from the association to the hidden unit, and also a modulatory link *b* (depicted by a blob) that runs from the hidden unit to the association. We assume that when the target CS → US increases in strength, this allows the *association* → *node* link *a* to activate the hidden unit via a standard associative mechanism. Any stimulus that is also present (such as the light in the figure) becomes bidirectionally associated with the node via normal classical conditioning processes (links *c* and *d* in Fig. 8); now when the light is presented it can activate the hidden unit via link *d*, which results in activation of the modulatory *node* → *association* link, *b*. This special modulatory link enables the light to act as an occasion setter, because this link allows *facilitation* of the association between the click and sucrose, without actually activating its components (click and sucrose). The incorporation of the hidden unit into this structure thus allows the occasion setter to influence the target association in a non-associative manner (via link *b*) while at the same time allowing the establishment of the occasion setter to obey normal associative principles (formation of link *d*) – as there is evidence that formation of occasion setters is subject to classic associative phenomena such as blocking (e.g. Bonardi, 1991).

This hierarchical account can explain the results of Experiments 1 and 3a; the more elements the transfer CS and US share with the association upon which the occasion setter acts, the greater its effect will be. It can also explain the results of the type reported by Bonardi and Jennings (2009), referred to in section 1. When the animals are initially trained on the biconditional discrimination *A*…*x*+, *A*…*y*−, *B*…*y*+, *B*…*x*−, *A* would become associated with the *x* → *food* and *y* → *no food* nodes, as described above. When *A* was then paired with shock, presentation of *A* would activate these two nodes, which would also become associated with shock. Thus when the *x* → *food* and *y* → *no food* pairings are presented at test, their nodes would be activated, which in turn would activate the shock representation to elicit the fear that was observed. It can also, as suggested above, accommodate a variety of the results that have been interpreted as unique evidence for the pseudo-configural theory of Honey and colleagues. For example, Close et al., 2009 trained animals on a discrimination in which *A* and *B* signalled that *x* would be reinforced (with food), and *C* and *D* that it would not, and also that *A* and *D* signalled that *y* would be reinforced, while *B* and *C* signalled that it would not (*Ax*+, *Bx*+, *Cx*−, *Dx*−, *Ay*+, *Dy*+, *By*−, *Cy*−). Then *A* was paired with shock and C with no shock; it was found that *Bx* elicited more fear than *Dx*. They argued that four different configural units would be recruited through this training; two of these would predict food, and have *ABx* and *ADy* as associates, and the other two would predict no food, and would be linked to *CDx* and *BCy*. The shock training would link the *ABx* and *ADy* units with shock, and the *CDx* and *BCy* with no shock. Thus activation of any unit linked to A would elicit fear, and any unit linked to C would allay it. Presenting *Bx* at test would provide two sources of activation to the *ABx* unit, while presenting *Dx* would provide one source of activation to *ABx* and *ADy*. As they argue that double activation of *ABx* is more effective than single activation of *ABx* and *ADy*, this produces the pattern of results obtained – more fear to *Bx* than to *Dx*. However, these results are equally consistent with the hierarchical account. This would posit that *A* and *B* are linked to the *x* → *food* association's hidden unit, *C* and *D* to the *x* → *no food* unit, *A* and *D* to the *y* → *food* unit and *B* and *C* to the *y* → *no food* unit. Pairing *A* with shock would associate the *x* → *food* and *y* → *food* hidden units with shock, and pairing *C* with no shock would associate the *x* → *no food* and *y* → *no food* units with shock absence. Presenting *B* alone would thus activate one association node which is paired with shock and one which is not, and the same would be true of *D*. However, the nonreinforced presentation of *x* on *Dx* trials would result in selective activation of the node linked to the *x* → *no food* association; this node, being associated with C and D, is associated with the absence of shock – resulting in less fear to *Dx* than to *Bx*.

In summary, standard configural theories cannot explain the results reported here, specifically the finding of more responding on same than on different trials in Experiments 1 and 3a. Moreover, although the pseudo-configural account succeeds where configural theories fail, it offers no explanation of why an opposite pattern of results, more responding on different trials, was observed in Experiment 4. A hierarchical structure of the type outlined above can, however, explain the entire pattern of results reported here, in addition to most of the findings previously regarded as unique report for the pseudo-configural account. But this should not be taken to imply that configural-type accounts are redundant; on the contrary, there is good evidence that with the right training conditions configural processes operate (e.g. Holland, 1989). A more conservative conclusion might therefore be that, when the training conditions favour a hierarchical rather than a configural solution, some process along the lines of that we have suggested here might predominate.

## Figures and Tables

**Table 1:** Design of Experiment 1.

Patterning training	Transfer CS training	Test	
		Same	Different
*A*: *x* → *suc*, *x*−, *A*−	*A*: *x* → *suc*, *x*−, *A*−	*A*: *w*	*A*: *z*
*B*: y → *oil*, *y*−, *B*−	*B*: *y* → *oil*, *y*−, *B*−	*B*: *z*	*B*: *w*
	*w* → *suc*, *z* → *oil*	*w* → *suc*	*z* → *oil*

*Note*: All stimuli were of 10 s duration, and a 5 s trace interval separated visual and auditory stimuli on compound trials. For details of counterbalancing see Sections 2.1.2 and 2.1.3.2.

**Table 2:** Design of Experiment 2.

Patterning training	Transfer CS and pseudo occasion setter training	Test	
		Same	Different
*A*: *x* → *suc*, *x*−, *A*−	*A*: *x* → *suc*, *x*−, *A*−	*C*: *x*	*C*: *y*
*B*: *y* → *oil*, *y*−, *B*−	*B*: y → *oil*, *y*−, *B*−	*D*: *y*	*D*: *x*
	*w* → *suc*, *z* → *oil*	*x* → *suc*	*y* → *oil*
	*C*… → *suc*, *D*… → *oil*		

*Note*: All stimuli were of 10 s duration, and a 5 s trace interval separated visual and auditory stimuli on compound trials; … denotes a 15-s trace interval before reinforcer delivery. For further details of counterbalancing see Sections 3.1.2 and 2.1.3.3.

**Table 3:** Design of Experiment 3.

Patterning training	Transfer CS and pseudo occasion setter training	Test 3a		Test 3b	
		Same	Different	Same	Different
*A*: *x* → *suc*, *x*−, *A*−	*A*: *x* → *suc*, *x*−, *A*−	*A*: *w*	*A*: *z*	*C*: *x*	C: *y*
*B*: *y* → *oil*, *y*−, *B*−	*B*: *y* → *oil*, *y*−, *B*−	*B*: *z*	*B*: *w*	*D*: *y*	*D*: *x*
	*w* → *suc*, *z* → *oil*	*w* → *suc*	*z* → *oil*	*x* → *suc*	*y* → *oil*
	*C*… → *suc*, *D*… → *oil*				

*Note*: All stimuli were of 10 s duration, and a 5 s trace interval separated visual and auditory stimuli on compound trials; … denotes a 15-s trace interval before reinforcer delivery. For further details of counterbalancing see Sections 4.1.3.2 and 4.1.3.3.

**Table 4:** Same and different test compounds in Experiment 4.

Test 1		Test 2		Test 3	
Same	Different	Same	Different	Same	Different
*C*: *x*	*C*: *y*	*C*: *w*	*C*: *z*	*w*: *x*	*z*: *y*
*D*: *y*	*D*: *x*	*D*: *z*	*D*: *w*	*z*: *y*	*w*: *x*

*Note*: All stimuli were of 10 s duration, and a 5 s trace interval separated the first and the second stimulus. For details of counterbalancing see Section 5.1.3.3.

## Footnotes

2 Although there have been a number of reports that occasion setters do not transfer to continuously reinforced transfer CSs (e.g. Rescorla, 1985), there is no corresponding evidence to suggest that the same is true of occasion setters (cf. Holland, 1989).
